# Subclinical Atherosclerosis, Vascular Risk Factors, and White Matter Alterations in Diffusion Tensor Imaging Findings of Older Adults With Cardiometabolic Diseases

**DOI:** 10.3389/fnagi.2021.712385

**Published:** 2021-08-20

**Authors:** Yoshiaki Tamura, Keigo Shimoji, Joji Ishikawa, Yoshinori Matsuo, So Watanabe, Hisae Takahashi, Shugo Zen, Aya Tachibana, Takuya Omura, Remi Kodera, Kazuhito Oba, Kenji Toyoshima, Yuko Chiba, Aya M. Tokumaru, Atsushi Araki

**Affiliations:** ^1^Department of Diabetes, Metabolism, and Endocrinology, Tokyo Metropolitan Geriatric Hospital, Tokyo, Japan; ^2^Department of Diagnostic Radiology, Tokyo Metropolitan Geriatric Hospital, Tokyo, Japan; ^3^Department of Cardiology, Tokyo Metropolitan Geriatric Hospital, Tokyo, Japan

**Keywords:** diffusion tensor imaging, atherosclerosis, brachial-ankle pulse wave velocity, intima-media thickness, ankle brachial index

## Abstract

White matter abnormalities may reflect cerebral microvessel disease. Diffusion tensor imaging (DTI) can help detect early changes in white matter integrity in each tract. However, studies investigating the relationship between subclinical atherosclerosis markers and white matter alterations in DTI findings are limited. This study aimed to examine associations between cardiovascular risk factors and indices of subclinical atherosclerosis—ankle brachial index (ABI), brachial-ankle pulse wave velocity (baPWV), and carotid artery intima-media thickness (IMT)—and altered white matter integrity in older patients. A total of 224 patients (aged ≥65 years) with cardiometabolic disease who underwent magnetic resonance imaging (MRI) and either plethysmography or cervical ultrasound at the start of the 3-year observational study period were included in this study. We measured fractional anisotropy (FA) and mean diffusivity (MD), which are indices of white matter integrity in seven white matter tracts. In a univariate analysis, lower ABI and higher baPWV values were associated with FA or MD abnormalities in several tracts, whereas IMT was scarcely associated with such change. In addition, high blood pressure and glycoalbumin/glycohemoglobin ratio (GA/HbA1c) and low body mass index (BMI) and triglyceride (TG) levels were associated with FA or MD abnormalities. In a multivariate analysis adjusted for age, sex, BMI, diastolic blood pressure, TG, and GA/HbA1c, the associations between ABI and FA or MD remained in all of either side of the following tracts: anterior thalamic radiation, forceps minor, inferior frontooccipital fasciculus (*p* < 0.001 for all) and superior longitudinal fasciculus (SLF; *p* < 0.05), whereas most of those between baPWV and FA or MD disappeared except for SLF (*p* < 0.05). These results indicate that low ABI could be an indicator of white matter abnormalities.

## Introduction

White matter fibers are susceptible to ischemia; white matter hyperintensity (WMH) in fluid-attenuated inversion recovery (FLAIR) images on magnetic resonance imaging (MRI) scans represent ischemic changes in cerebral microvessels (Matsusue et al., 2006). In addition, an increase in the volume of WMH is associated with cognitive dysfunction and functional disability (Debette and Markus, 2010; Kreisel et al., 2013).

Cardiovascular risk factors, including hypertension and hyperglycemia, are associated with the progression of white matter abnormality. Our previous study has shown that the ratio of glycoalbumin and glycohemoglobin (GA/HbA1c), an indicator of glycemic variability, is associated with WMH volume in older patients with diabetes (Tamura et al., 2017).

Diffusion tensor imaging (DTI) is frequently used to evaluate white matter integrity. DTI is a technique in which multiple-directional diffusion-weighted MRI images are reconstructed, and fractional anisotropy (FA, alterations of anisotropy) or mean diffusivity (MD, diffusivity of water molecule) values are used as indices of damage to the white matter. This method has several advantages: it can detect slight alterations in the white matter earlier than conventional MRI imaging, and it can detect these alterations separately in each tract (Assaf and Pasternak, 2008), allowing for the correlation of the damage observed in particular tracts with disease presentation and progression. We have previously reported findings from a cross-sectional study, showing that abnormalities of white matter integrity in the left anterior thalamic radiation (ATR) and right inferior frontooccipital fasciculus (IFOF) are associated with sarcopenia and its correlates (Tamura et al., 2021).

In addition to white matter fibers being susceptible to ischemia, markers of atherosclerosis have been associated with the severity of WMH lesions. The Rotterdam Study has shown that common carotid artery (CCA) intima-media thickness (IMT), carotid plaques, and the ankle brachial index (ABI) are associated with WMH (Bots et al., 1993). Other studies reported that carotid-femoral pulse wave velocity (cfPWV; Coutinho et al., 2011) or brachial-ankle pulse wave velocity (baPWV; Saji et al., 2011) may affect the severity of WMH. However, there have been few reports showing that the loss of white matter integrity indicated by DTI findings is associated with markers of atherosclerosis. Badji et al. (2019) and Wei et al. (2020) reported that cfPWV is associated with some DTI indices that may suggest white matter abnormalities in various cerebral lesions; meanwhile, Han et al. (2021) have shown that high baPWV is associated with the deterioration of DTI markers. Nevertheless, few studies have examined whether any particular measure of subclinical atherosclerosis is specifically associated with early white matter changes, as indicated by DTI. Moreover, little is known about any associations between cardiovascular risk factors and alterations in white matter integrity.

Thus, in this study, we investigated whether multiple indices of subclinical atherosclerosis (ABI, carotid artery IMT, and baPWV) or cardiovascular risk factors are associated with changes in DTI values of the white matter tracts in older patients with cardiometabolic diseases. We further investigated the following problems: whether the associations between the atherosclerotic index and white matter abnormalities are observed independent of cardiovascular risk factors, which atherosclerotic index is the most predictable for the loss of white matter integrity, and whether the influences are observed in specific white matter tracts.

## Materials and Methods

Data from older patients (aged ≥65 years), who visited the Frailty Clinic in Tokyo Metropolitan Geriatric Hospital for the evaluations of frailty, cognitive function, and cardiovascular disease in the prospective study by Tamura et al. (2018) were included. Most patients were treated at the Departments of Diabetes and Cardiology and had cardiometabolic diseases, such as hypertension, diabetes mellitus, and dyslipidemia. The patients’ diagnoses and smoking history were extracted from their medical records. Of 284 patients who underwent baseline brain MRI scanning, details of which are outlined elsewhere (Tamura et al., 2021), data from 224 patients who underwent examination of subclinical atherosclerosis were used for analyses. Of these 224 patients, 211 and 130 of them underwent plethysmography and cervical ultrasound respectively, whereas 117 of them underwent both. The ethics committee of the Tokyo Metropolitan Geriatric Hospital approved this study. All participants provided written informed consent in accordance with the Declaration of Helsinki.

### MRI Acquisition

MRI acquisition was performed as previously described (Tamura et al., 2021), using a Discovery MR750w 3.0 MRI system with a 16-channel head coil (General Electric Healthcare, Milwaukee, WI, USA). DTI was performed in the axial plane along the anterior commissure—posterior commissure line, using single shot spin-echo echoplanar imaging sequence and gradient encoding schemes with 32 non-collinear directions acquired at a b-value of 1,000 s/mm^2^ and with 1 non-diffusion-weighted image at a b-value of 0 s/mm^2^. Other parameters were set as follows: repetition time, 17,000 ms; echo time, 95.7 ms; the number of excitations, 1; slices, 66, without gaping; voxel size, 1 × 1 × 2 mm^3^; field of view, 256 mm; matrix size, 256 × 256.

### DTI Analysis

The DTI analysis was also performed as previously described (Tamura et al., 2021), using the Oxford University FMRIB Software Library. In this study, we selected seven white matter tracts as regions of interest (shown in Supplementary Table 1). We created mean FA and MD skeletons in all tracts to represent the center of each tract by applying the non-near registration of all images to the MNI152 standard space. Subsequently, the FA and MD data of each subject were spatially normalized to the mean skeleton, and the FA and MD values of each tract were calculated and used as the indices of white matter abnormality. FA indicates the strength of anisotropy of water molecules along the long axis direction of neuron, and its values ranged from 0 to 1; lower values represented greater deterioration of tract integrity. In contrast, higher MD values represented greater impairment of white matter integrity.

It has been shown that the values of FA and MD vary considerably widely between the gray/white matter and among white matter tracts (Pierpaoli et al., 1996), but it has been shown that it is meaningful to compare the values of FA or MD between some given conditions in the same tracts (Reijmer et al., 2013).

### Evaluation of Subclinical Atherosclerosis

ABI and baPWV were measured using a pulse waveform analyzer: BP-203RPE (Omron Healthcare Company Limited, currently manufactured by Fukuda Colin Company Limited, Tokyo, Japan). ABI was calculated by dividing the ankle blood pressure by the brachial blood pressure (whichever side was higher). To obtain baPWV, the difference in distance from the ostium aortae to the ankle and from the ostium aortae to the upper arm was first estimated from body height. Subsequently, baPWV was calculated by dividing this difference by the time gap between the pulse wave in the upper arm and ankle. ABI value < 0.90 indicates the presence of peripheral arterial disease, and in general, baPWV >1,800 cm/s indicated high-risk group for cardiovascular events even if ABI is higher than 1.0, although its values are affected by aging (Tomiyama et al., 2016). The smaller ABI values of the left/right sides (ABImin), and greater baPWV values of the left/right sides (PWVmax) were used for analysis.

IMT, carotid artery plaques, and stenosis were measured using ultrasonography (either of the following machines: FX-75 and ARIETTA 70, Hitachi-Aloka, Company Limited, currently manufactured by Hitachi, Company Limited, Tokyo, Japan, Aplio500 and Aplio300, Toshiba, Company Limited, Tokyo, Japan, LOGIQ E9, GE Healthcare Japan, Company Limited, Tokyo, Japan). IMT was defined as the total thickness of the tunica intima and tunica media in the CCA. The maximum values of IMT observed on both sides were defined as IMTmax. Stenosis in either of CCA and/or internal carotid artery more than 40% in area ratio method was defined as carotid artery stenosis. It is known that the progression of IMT predicts cardiovascular events well, but its value is also affected by age. Engelen et al. (2013) established a normal range of IMT in CCA in healthy population; according to the report, the mean and standard deviation of IMT at 79 years of age (median age of our study) is 0.734 and 0.129 mm for men and 0.714 and 0.119 mm for women, respectively.

It has been reported that reproducibility in the measurement of subclinical atherosclerosis is highly reliable; in previous reports, the intraclass correlation coefficient for the interobserver reproducibility of AB I (Ichihashi et al., 2020), baPWV (Meyer et al., 2016), and IMTmax (Martínez et al., 2015) were shown as 0.96, 0.84, and 0.69–0.86. As evaluations of these subclinical variables of atherosclerosis were done by laboratory engineers whereas the evaluations of DTI variables were done by a radiologist (K.S.), these outcomes were mutually blinded.

### Statistical Analysis

First, correlations between ABImin, PWVmax, IMTmax, and FA and MD values of each white matter tract were evaluated using the Spearman rank correlation test. Correlations were also evaluated between DTI values and other metabolic markers, such as smoking status (Brinkman Index), blood pressure, body mass index (BMI), HbA1c, low-density lipoprotein cholesterol (LDL-C), high-density lipoprotein cholesterol (HDL-C), GA/HbA1c, and physical activity (Craig et al., 2003).

Next, multiple linear regression analyses were performed using FA or MD values of each tract as dependent variables and ABImin, PWVmax, and IMTmax values as explanatory variables, with adjustments for potential confounders, specifically, age, sex, BMI, diastolic blood pressure, and GA/HbA1c. Comparisons between values were performed with the Mann-Whitney test for continuous variables; the chi-square test was used for categorical variables. Statistical significance was set at a *P*-value of <0.05. All analyses were performed using SPSS ver.20 (IBM Corp., Armonk, NY, United States).

## Results

The baseline characteristics of the participants are presented in Table 1. The prevalence of diabetes, hypertension, and dyslipidemia were 52%, 80%, and 67%, respectively. The median MMSE score was 28 points. The median ABImin, baPWVmax, and IMTmax values were 1.12, 1,856 cm/s, and 1.3 mm, respectively. Only 6.6% of patients had an ABI value of <0.9, an indicator of peripheral artery disease (PAD). Ninety-two percent (120/130) had carotid artery plaques and 16% (21/130) had carotid artery stenosis.

**Table 1:** Clinical characteristic of subjects.

	Total (*n* = 224)
Age (years)	79 [75–83]
Women (%)	62.9
BMI (kg/m^2^)	22.6 [20.7–25.3]
Hypertension (%)	79.5
Smoking (Current/Past/Never; %; *n* = 221)	4/38/58
Diabetes Mellitus (%)	51.8
Dyslipidemia (%)	67.0
Cardiovascular disease (%; *n* = 221)	15.4
sBP (mmHg)^†^	131 [122–140]
dBP (mmHg)^†^	73 [67–81]
Alb (g/dl)	4.0 [3.8–4.2]
Hb (g/dl)	12.9 [12.0–13.8]
HbA1c (%; *n* = 222)	6.2 [5.8–6.9]
GA/HbA1c (*n* = 204)	2.74 [2.58–2.97]
TG (mg/dl; *n* = 223)	109 [80–154]
LDL-C (mg/dl; *n* = 223)	109 [90–130]
HDL-C (mg/dl; *n* = 223)	57 [48–68]
Creatinine (mg/dl)	0.82 [0.69–1.03]
eGFR (ml/min/1.73 m^2^)	57.0 [47.3–66.8]
MMSE (*n* = 223)	28.0 [26.0–29.0]
EE (Mets • min/week; *n* = 220)	1193 [503–2258]
FA (whole brain)	0.49 [0.48–0.50]
MD (whole brain; x10^−3^)	0.77 [0.75–0.79]
ABImin (*n* = 211)	1.12 [1.06–1.17]
baPWVmax (cm/s; *n* = 211)	1856 [1622–2088]
IMTmax (mm; *n* = 130)	1.3 [1.1–1.6]
Carotid artery stenosis (≥40%; %; *n* = 130)	16

*Median [25–75% tile]. BMI, body mass index; sBP, systolic blood pressure; dBP, diastolic blood pressure; Alb, albumin; Hb, hemoglobin; GA, Glycoalbumin; TG, triglyceride; LDL-C, low-density lipoprotein cholesterol; HDL-C, high-density lipoprotein cholesterol; Cre, creatinine; eGFR, estimated glomerular filtration rate; MMSE, mini-mental state examination; EE, energy expenditure; FA, fractional anisotropy; MD, mean diffusivity; ABI, ankle brachia index; baPWV, brachial-ankle pulse wave velocity; IMT, intima-media thickness. Information of some items lacked in some patients; these items are shown as (n = number of samples)*.

**Figure 1:**
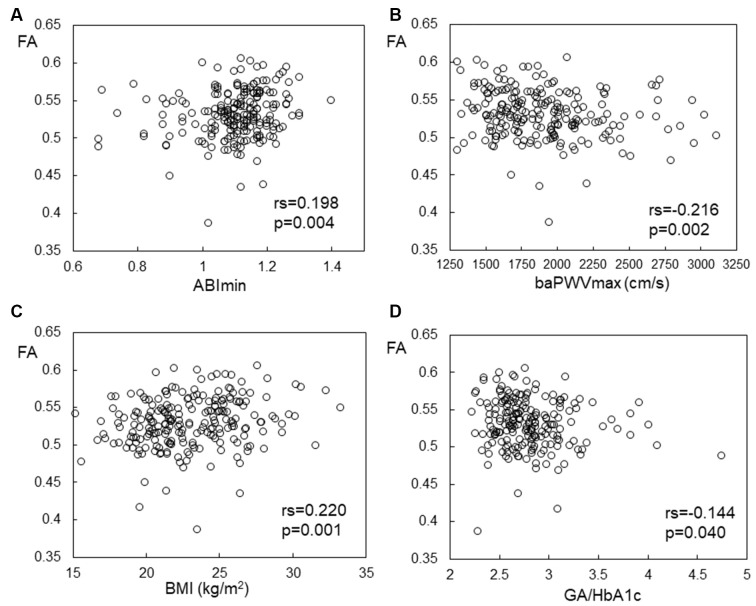
Correlation between FA in the left ATR and **(A)** ABImin, **(B)** baPWVmax, **(C)** BMI, and **(D)** GA/HbA1c. FA, fractional anisotropy; ATR, anterior thalamic radiation; ABI, ankle brachia index; baPWV, brachial-ankle pulse wave velocity; BMI, body mass index; GA/HbA1c, glycoalbumin/glycohemoglobin ratio.

The Spearman correlation coefficients for associations between the indices of subclinical atherosclerosis (ABImin, baPWVmax, and IMTmax) and FA or MD values in the white matter tracts are shown in Table 2. ABImin values were positively and baPWVmax values were negatively associated with FA, and ABImin values were negatively and baPWVmax values were positively associated with MD in almost all the tracts. The associations between FA in the left ATR with ABImin and baPWVmax are shown in Figures 1A,B. In contrast, IMTmax was not associated with DTI values in almost all the tracts.

**Table 2:** Spearman correlations of ABImin, baPWVmax, and IMTmax with FA and MD in seven white matter tracts.

		FA							MD						
		lATR	rATR	FM	lIFOF	rIFOF	lSLF	rSLF	lATR	rATR	FM	lIFOF	rIFOF	lSLF	rSLF
ABImin	*r*_s_	**0.198**	0.116	**0.176**	**0.204**	**0.214**	**0.217**	**0.188**	**−0.198**	−0.120	**−0.140**	**−0.135**	**−0.163**	**−0.169**	**−0.170**
(*n* = 211)	*p*	0.004**	0.092	0.011*	0.003**	0.002**	0.001**	0.006**	0.004**	0.082	0.042*	0.050*	0.018*	0.014*	0.013*
baPWVmax	*r*_s_	**−0.216**	**−0.219**	**−0.206**	**−0.237**	**−0.228**	**−0.192**	**−0.137**	**0.209**	**0.139**	**0.190**	**0.254**	**0.213**	**0.235**	**0.184**
(*n* = 211)	*p*	0.002**	0.001**	0.003**	0.001**	0.001**	0.005**	0.047*	0.002**	0.044*	0.006**	<0.001**	0.002**	0.001**	0.007**
IMT max	*r*_s_	0.011	−0.037	−0.049	−0.070	−0.081	−0.055	0.013	0.132	**0.180**	0.112	0.092	0.137	0.118	0.097
(*n* = 130)	*p*	0.899	0.677	0.578	0.432	0.360	0.537	0.882	0.135	0.040*	0.205	0.297	0.121	0.183	0.272

FA, fractional anisotropy; MD, mean diffusivity; ABI, ankle brachia index; baPWV, brachial-ankle pulse wave velocity; IMT, intima-media thickness; lATR/rATR, left/right anterior thalamic radiation; FM, Forceps minor; lIFOF/rIFOF, left/right inferior frontooccipital fasciculus; lSLF/rSLF, right/left superior longitudinal fasciculus; rs, Spearman’s correlation coefficient. *p < 0.05, **p < 0.01. Bold values indicate *p* < 0.05.

The Spearman correlation coefficients for the associations between cardiovascular risk factors and FA or MD values in the white matter tracts are shown in Supplementary Table 2 BMI was positively associated with FA in bilateral ATRs (The relationship between BMI and FA values of the left ATR are shown in Figure 1C). GA/HbA1c showed strong negative and positive associations with FA and MD, respectively, in all tested tracts, except for the left superior longitudinal fasciculus (SLF; The relationship between GA/HbA1c and FA values of the left ATR are shown in Figure 1D), whereas HbA1c showed no significant correlation with either of FA and MD. Triglyceride (TG) levels showed either positive associations with FA or negative associations with MD in all tested tracts. Diastolic blood pressure showed a significant correlation with FA in the bilateral SLF. Physical activity was positively correlated with MD only in the right ATR. However, other atherosclerotic risk factors, smoking status (Brinkman index), or LDL-C or HDL-C levels showed no correlation with the DTI indices.

Table 3 shows the results of multiple regression analysis with FA or MD values of each tract set as dependent variables and ABImin and baPWVmax values set as explanatory variables. After adjustment for covariates (model 1: age and sex, model 2: model 1 plus BMI and diastolic blood pressure, model 3: model 2 plus TG and GA/HbA1c), the associations between ABImin and FA and MD values remained significant in most of the tested tracts, except for the right SLF. However, after adjusting for confounding variables, the associations between baPWVmax and FA and MD values were attenuated in most of the tested tracts, except for the left SLF.

**Table 3:** Multiple linear regression analyses for the association of ABImin, baPWVmax with FA and MD in seven white matter tracts.

		FA							MD						
		lATR	rATR	FM	lIFOF	rIFOF	lSLF	rSLF	lATR	rATR	FM	lIFOF	rIFOF	lSLF	rSLF
(A) ABImin
Model 1	β	**0.141**	0.030	**0.217**	**0.197**	**0.194**	0.126	0.087	**−0.174**	−0.125	**−0.229**	−0.127	**−0.160**	**−0.110**	**−0.147**
	*p*	0.031*	0.160	0.001**	0.003**	0.004**	0.063^¶^	0.195	0.010*	0.062^¶^	<0.001**	0.063	0.018*	0.101	0.026*
Model 2	β	**0.169**		**0.229**	**0.217**	**0.212**	**0.134**		**−0.185**	−0.128	**−0.239**	**−0.142**	**−0.169**		**−0.155**
	*p*	0.009**	<0.001**	0.001**	0.001**	0.047*		0.006**	0.059^¶^	<0.001**	0.038*	0.013*		0.020*
Model 3	β	**0.169**		**0.194**	**0.185**	**0.179**	**0.144**		**−0.208**	**−0.144**	**−0.203**	−0.121	**−0.150**		−0.126
	*p*	0.011*		0.004**	0.007**	0.010*	0.037*		0.003**	0.045*	0.003**	0.093^¶^	0.038*		0.074^¶^
(B) baPWVmax
Model 1	β	**−0.142**	−0.126	−0.088	−0.122	−0.111	**−0.155**	−0.038	0.108	0.034	0.032	0.128	0.089	**0.174**	0.041
	*p*	0.037*	0.065^¶^	0.192	0.077^¶^	0.110	0.028*	0.580	0.122	0.629	0.635	0.069^¶^	0.205	0.012*	0.547
Model 2	β	−0.099	−0.109		−0.085		−0.120					0.098		**0.140**	
	*p*	0.149	0.124		0.228		0.093^¶^					0.175		0.046*	
Model 3	β						**−0.158**							**0.179**	
	*p*						0.030*							0.015*	

Model 1: adjusted for age and gender. Model 2: Model 1 + BMI and diastolic blood pressure. Model 3: Model 2 + TG and GA/HbA1c. FA, fractional anisotropy; MD, mean diffusivity; ABI, ankle brachia index; baPWV, brachial-ankle pulse wave velocity; lATR/rATR, left/right anterior thalamic radiation; FM, Forceps minor; lIFOF/rIFOF, left/right inferior frontooccipital fasciculus; lSLF/rSLF, right/left superior longitudinal fasciculus; BMI, body mass index; TG, triglyceride; GA/HbA1c, glycoalbumin/glycohemoglobin ratio. *p < 0.05, **p < 0.01, ^¶^*p* < 0.1, *p* < 0.05. Bold values indicate *p* < 0.05.

For another index for carotid artery lesion, we tested the association between carotid artery stenosis and DTI values, since almost all patients had atherosclerotic plaques. Multiple regression analysis showed that carotid artery stenosis had a significant association with MD value in FM, and a tendency to associate with MD value in the right IFOF (β = 0.186, *p* = 0.034 / β = 0.163, *p* = 0.082, respectively).

## Discussion

This study aimed to investigate the association between white matter changes evaluated using DTI and the indices of subclinical atherosclerosis. The present findings suggest that ABImin is associated with alterations in white matter integrity in the left ATR, forceps minor, and right IFOF. Several studies have shown associations between arterial stiffness and disruption of white matter integrity, as evaluated by DTI. Badji et al. (2019) reported that cfPWV was associated with some DTI indices in four white matter tracts, including the corpus collosum and SLF in older subjects. In addition, Wei et al. (2020) have shown significant associations between cfPWV and FA and MD values in the whole cerebrum and various lesions in the regions of interest divided in each lobe and the anterior/posterior corpus callosum. More recently, Han et al. (2021) has shown an association between baPWV and DTI indices, including FA and MD, calculated for the whole brain in middle-aged subjects. However, the present study is the first to demonstrate that a low ABI value is associated with the loss of white matter integrity in specific tracts.

Various cardiovascular risk factors are known to be involved in the formation of white matter lesions. In their review, Wassenaar et al. (2019) showed that hypertension, obesity, diabetes, and smoking, all of which are risk factors for atherosclerosis, are also associated with the loss of white matter integrity detected by DTI. In contrast, in our previous study, we have shown that low BMI, low diastolic blood pressure, and high GA/HbA1c are associated with the total volume of cerebral WMH in patients with diabetes mellitus (Tamura et al., 2017). In this study, we found similar correlations between low BMI, GA/HbA1c, and white matter changes; GA/HbA1c was particularly strongly associated with DTI values in all the tested tracts, whereas HbA1c was not. This could be attributed to the fact that GA/HbA1c is associated with glucose variability, which induces higher oxidative stress and vascular endothelial dysfunction (Torimoto et al., 2013) could damage the white matter.

Although we have previously shown a reversed association between diastolic blood pressure and WMH volumes, herein, we observed a weak correlation between high diastolic blood pressure and disturbed integrity of white matter. This finding may be explained by the fact that in the stage where MRI-visible WMHs are formed, low blood pressure is a risk factor due to the decline of perfusion rate in small arteries. Meanwhile, the stage where changes in FA or MD could be detected occurs earlier; in this MRI-detectable stage, high blood pressure, a risk factor for atherosclerosis, may have a greater detrimental effect. The direction of this association with BMI is opposite to that in the review by Wassenaar et al.; this discrepancy may be due to the fact that the studies included in this review focused on a considerably younger population. For older adults, including the present study participants, low BMI, which may reflect malnutrition, may also be a strong risk factor for white matter damage (de van der Schueren et al., 2016).

In the present study, higher TG levels seemed protective against damage to white matter integrity. Higher TG levels are associated with insulin resistance, which may accelerate atherosclerosis; however, previous findings have been conflicting. For example, Iriondo et al. (2021) have shown that higher TG and LDL-C levels and lower HDL-C levels were risk factors for alteration of white matter microstructure. In contrast, Guoxiang et al. (2018) have shown that TG levels were negatively associated with the degree of WMH, a finding similar to that of the present study. This association may be accounted for just like BMI; in older patients, TG levels may indicate the state of nutrition rather than insulin resistance. The association between cardiovascular risk factors and white matter changes suggests that disturbed white matter integrity detected by DTI could be modifiable and treatable by optimal control of postprandial hyperglycemia, blood pressure, and hypertriglyceridemia and prevention of malnutrition.

The reason why the association between ABImin and FA and MD values was stronger than that with baPWVmax remains unclear. Some explanations could be made. First, baPWV has a stronger correlation with chronological age and blood pressure level than ABI. Second, the relevance of PWV could differ between different regions. In most Japanese institutions, PWV is measured as baPWV, which is quite easily performed. It had previously been shown that among various methods of PWV measurement, cfPWV is the most clinically relevant index reflecting aortic stiffness, and PWV measured outside the aortic track, such as baPWV, is less predictive for a cardiovascular event (Laurent et al., 2006). However, many recent reports from east Asia, including Japan, have shown that baPWV is also relevant for predicting cardiovascular death or events (Munakata, 2016; Tomiyama et al., 2016), suggesting this could be another good indicator of arterial stiffness. Third, PWV values may be inaccurate in patients with severe PAD (Aboyans et al., 2016); however, this limitation is unlikely to affect the present findings, as the number of patients with ABI of <0.9 was small. Del Brutto et al. (2015) reported that a degree of WMH was associated with ABI abnormality, but this association was observed in patients with ABI values of <0.9 or those of ≥1.4, which indicate the presence of PAD. The relevance of ABI decline within the normal range remains to be elucidated. In our study, most ABI values were in the range of 0.9–1.4, indicating that a slight ABI decrease before the presentation of PAD may increase the risk of white matter abnormalities. When we set the cutoff level of ABI to 1.0, patients with ABI of < 1.0 showed significantly lower FA and higher MD values in most tracts, except for the bilateral ATR (data not shown). Although there are few reports of associations between low-normal ABI values and the incidence of cardiovascular disease, a meta-analysis showed that the risk of cardiovascular mortality was higher even in the ABI 0.9–1.0 group than in the 1.1–1.2 reference group (Fowkes et al., 2008). Furthermore, Ishizuka et al. (2014) reported that the risk of low ABI for neurologic degeneration in ischemic stroke was similarly high when setting a lower limit of ABI, either 0.9 or 1.0. These reports and our results indicate that ABI, an index for arteriosclerosis in the lower extremity, actually is associated with an atherosclerotic lesion in the brain, even if the change is subtle. Further studies are needed to clarify the mechanism of a decline in ABI values on the change of white matter integrity.

In the present study, there was no association between IMTmax and DTI indices, although the small sample size may preclude the detection of this difference. This finding is consistent with that of Shenkin et al. (2010) who showed that IMT was not associated with FA or MD values in healthy older subjects. However, these authors have also shown that white matter changes were significantly correlated with maximum carotid stenosis, which may indicate large vessel disease. Additionally, there have been two meta-analytic studies showing a positive association between carotid artery stenosis and WMH (Ye et al., 2018) and some DTI indices (Baradaran et al., 2016). This was applicable to our samples; when we took carotid artery stenosis into account, stenosis was partially associated with MD in several tracts, showing that it could be another indicator for DTI abnormality. However, our results indicate that ABI values may reflect small vessel disease in the brain more accurately than IMT and earlier than carotid artery stenosis.

In the multiple regression analysis, ABImin was correlated with FA and MD values in the broad cerebral areas, except for SLF. The tracts examined in this study have various important roles; ATRs are involved in executive function (Biesbroek et al., 2013) and IFOFs are involved in visuospatial cognition (Poole et al., 2018). SLFs are also involved in executive function, as are ATRs, and damage to these tracts is associated with slower gait (Poole et al., 2018). Forceps minor is a set of tracts that spread from the corpus callosum to the cortex of the occipital lobe and is closely related to the function of the corpus callosum. The corpus callosum is a tract connecting the hemispheres, associated with executive function (Zheng et al., 2014). Early detection of ABI decline and white matter alteration and optimal control of cardiovascular risk factors may help retain a broad range of cognitive function and functional ability over time.

This study has several limitations. First, this was a cross-sectional study, precluding conclusions on causality; however, given the pathophysiology, it is plausible that white matter changes may result from the acceleration of atherosclerotic lesions. Future longitudinal observational studies should examine how alterations in each white matter tract affect the cognitive function or functional ability. Second, the backgrounds of the subjects are quite heterogeneous. Most participants in our study had cardiometabolic diseases, and some of them overlapped, suggesting that they may be more susceptible to atherosclerotic diseases than community-dweling older adults. With participants recruited from the general population, study findings may be different. Moreover, this study was performed at a single Japanese institution; future multicenter and epidemiological studies are required to validate the present findings. Finally, as we describe in the Methods and Discussion sections above, we could evaluate only limited indices for subclinical atherosclerosis, and the associations between DTI values and uninvestigated indices, such as cfPWV remain to be clarified.

In conclusion, the low ABI of the present study, an index of peripheral arteriosclerosis, was associated with alterations in the integrity of various white matter tracts. Further studies are needed to clarify the influence of these changes on the pathophysiology of cognitive dysfunction and frailty and, subsequently, to clarify the effect of cardiovascular risk factor management on the prevention of white matter alterations and geriatric syndrome onset.

## Data Availability Statement

The datasets presented in this article are not readily available because we do not agree for our participants’ data to be shared publicly, so supporting data is not available. Requests to access the datasets should be directed to Yoshiaki Tamura tamurayo@tmghig.jp.

## Ethics Statement

The studies involving human participants were reviewed and approved by The ethics committee of the Tokyo Metropolitan Geriatric Hospital. The patients/participants provided their written informed consent to participate in this study.

## Author Contributions

YT and AA designed the study, analyzed the data, and wrote the draft of the manuscript. KS contributed to DTI data collection. JI, YM, SW, HT, SZ, ATa, RK, KO, KT, and YC contributed to clinical data collection, analysis, and interpretation of data. JI, TO, and ATo contributed to data interpretation and critically reviewed the manuscript. All authors contributed to the article and approved the submitted version.

## Conflict of Interest

The authors declare that the research was conducted in the absence of any commercial or financial relationships that could be construed as a potential conflict of interest.

## Publisher’s Note

All claims expressed in this article are solely those of the authors and do not necessarily represent those of their affiliated organizations, or those of the publisher, the editors and the reviewers. Any product that may be evaluated in this article, or claim that may be made by its manufacturer, is not guaranteed or endorsed by the publisher.
