# Supplementary material for: Subclinical Atherosclerosis, Vascular Risk Factors, and White Matter Alterations in Diffusion Tensor Imaging Findings of Older Adults With Cardiometabolic Diseases
Source: Front Aging Neurosci. 2021 Aug 20;13:712385. doi: 10.3389/fnagi.2021.712385 (PMC8417784; doi:10.3389/fnagi.2021.712385)
Supplement: Supplementary file 1 [file Table_1.pdf]

Supple Table 1 White matter tracts investigated as regions-of-interest

Left/right Anterior thalamic radiation (ATR)

Forceps minor (FM)

Left /right inferior frontooccipital fasciculus (IFOF)

Left /right superior longitudinal fasciculus (SLF)

Supple Table 2. Spearman correlations of cardiovascular risk factors with FA and MD in seven white matter tracts

		FA							MD						
		lATR	rATR	FM	lIFOF	rIFOF	lSLF	rSLF	lATR	rATR	FM	lIFOF	rIFOF	lSLF	rSLF
Age	r_s	**-0.193**	**-0.269**	**-0.348**	**-0.291**	**-0.281**	**-0.159**	**-0.257**	**0.242**	**0.244**	**0.415**	**0.248**	**0.255**	**0.298**	**0.337**
	p	**0.004****	**<0.001****	**<0.001****	**<0.001****	**<0.001****	**0.017***	**<0.001****	**<0.001****	**<0.001****	**<0.001****	**<0.001****	**<0.001****	**<0.001****	**<0.001****
BMI	r_s	**0.220**	**0.155**	0.069	0.088	0.105	-0.046	0.021	**-0.143**	-0.079	-0.065	-0.052	-0.041	0.015	0.001
	p	**0.001****	**0.020***	0.307	0.188	0.116	0.489	0.749	**0.033***	0.238	0.334	0.439	0.546	0.819	0.992
Brinkman Index	r_s	0.065	0.010	0.019	-0.095	-0.091	0.015	0.050	-0.011	0.023	0.017	0.088	0.051	0.051	0.043
(n=207)	p	0.351	0.890	0.784	0.173	0.194	0.829	0.472	0.877	0.737	0.807	0.206	0.464	0.466	0.539
sBP	r_s	-0.077	0-.032	-0.076	-0.123	-0.122	**-0.161**	**-0.192**	0.036	-0.015	0.057	0.120	0.091	0.119	0.060
	p	0.254	0.637	0.254	0.066	0.068	**0.016***	**0.004****	0.587	0.823	0.399	0.074	0.177	0.076	0.370
dBP	r_s	-0.117	0-.004	-0.029	0-.074	-0.048	**-0.167**	**-0.141**	0.053	-0.076	-0.012	0.079	0.006	0.096	0.025
	p	0.081	0.948	0.670	0.273	0.475	**0.012***	**0.036***	0.428	0.258	0.854	0.241	0.923	0.151	0.712
LDL-C	r_s	-0.017	0.023	0.038	0.008	0.046	-0.039	0.013	0-.056	**-0.142**	-0.066	0.010	-0.086	0.010	0-.093
(n=223)	p	0.805	0.731	0.575	0.903	0.491	0.557	0.851	0.403	**0.034***	0.328	0.879	0.199	0.885	0.166
HDL-C	r_s	-0.068	-0.079	0.008	-0.042	-0.066	-0.066	-0.059	-0.001	-0.015	-0.074	0.032	0.038	0.053	0-.010
(n=223)	p	0.310	0.242	0.905	0.530	0.323	0.324	0.381	0.990	0.827	0.269	0.639	0.571	0.431	0.884
TG	r_s	**0.141**	**0.187**	**0.151**	**0.133**	**0.161**	**0.147**	0.127	-0.121	**-0.150**	-0.108	-0.071	**-0.145**	-0.130	**-0.148**
(n=223)	p	**0.036***	**0.005****	**0.024***	**0.047***	**0.016***	**0.028***	0.058	0.071	**0.025***	0.109	0.289	**0.031***	0.053	**0.027***
HbA1c	r_s	0.046	0.031	0-.069	-0.092	-0.060	0.046	0.028	0.011	-0.006	0.005	-0.031	-0.048	-0.015	-0.010
(n=222)	p	0.499	0.644	0.304	0.174	0.376	0.500	0.681	0.875	0.926	0.940	0.641	0.473	0.819	0.882
GA/HbA1c	r_s	**-0.144**	**-0.223**	**-0.224**	**-0.278**	**-0.267**	-0.080	**-0.157**	**0.179**	**0.219**	**0.175**	**0.160**	**0.185**	0.103	**0.221**
(n=204)	p	**0.040***	**0.001****	**0.001****	**<0.001****	**<0.001****	0.253	**0.025***	**0.011***	**0.002****	**0.012***	**0.023***	**0.008****	0.143	**0.002****
Physical Activity	r_s	0.049	0.061	0.044	0.055	0.102	0.053	0.070	-0.123	**-0.156***	-0.099	-0.045	-0.095	-0.096	-0.074
(n=220)	p	0.473	0.369	0.518	0.420	0.131	0.432	0.300	0.068	**0.020***	0.144	0.509	0.158	0.157	0.276

FA: fractional anisotropy, MD: mean diffusivity , ATR: anterior thalamic radiation, FM; Forceps minor, IFOF: inferior frontooccipital fasciculus, SLF: superior longitudinal fasciculus BMI: body mass index, sBP: systolic blood pressure, dBP: diastolic blood pressure, TG: triglyceride, GA: glycoalbumin, HbA1c: glycohemoglobin, r_s: Spearman's correlation coefficient **p<0.01, *p<0.05 Bold values indicate p<0.05.
